# Mating strategy is determinant of adenovirus prevalence in European bats

**DOI:** 10.1371/journal.pone.0226203

**Published:** 2020-01-07

**Authors:** Federica Rossetto, Maria Iglesias-Caballero, H. Christoph Liedtke, Ivan Gomez-Mestre, Jose M. Berciano, Gonzalo Pérez-Suárez, Oscar de Paz, Carlos Ibáñez, Juan E. Echevarría, Inmaculada Casas, Javier Juste

**Affiliations:** 1 Evolutionary Biology Unit, Estación Biológica Doñana (CSIC), Sevilla, Spain; 2 National Center of Microbiology, (ISCIII), Madrid, Spain; 3 Department of Life Sciences, University of Alcalá, Alcalá de Henares, Madrid, Spain; 4 CIBER Epidemiology and Public Health (CIBERESP), Madrid, Spain; University of Pretoria, SOUTH AFRICA

## Abstract

Adenoviruses are double-strained DNA viruses found in a great number of vertebrates, including humans. In order to understand their transmission dynamics, it is crucial, even from a human health perspective, to investigate how host traits influence their prevalence. Bats are important reservoirs for adenoviruses, and here we use the results of recent screenings in Western Europe to evaluate the association between characteristic traits of bat species and their probability of hosting adenoviruses, taking into account their phylogenetic relationships. Across species, we found an important phylogenetic component in the presence of adenoviruses and mating strategy as the most determinant factor conditioning the prevalence of adenoviruses across bat species. Contrary to other more stable mating strategies (e.g. harems), swarming could hinder transmission of adenoviruses since this strategy implies that contacts between individuals are too short. Alternatively, bat species with more promiscuous behavior may develop a stronger immune system. Outstandingly high prevalence of adenoviruses was reported for the Iberian species *Pipistrellus pygmaeus*, *P*. *kuhlii* and *Nyctalus lasiopterus* and we found that in the latter, males were more likely to be infected by adenoviruses than females, due to the immunosuppressing consequence of testosterone during the mating season. As a general trend across species, we found that the number of adenoviruses positive individuals was different across localities and that the difference in prevalence between populations was correlated with their geographic distances for two of the three studied bat species (*P*. *pygmaeus* and *P*.*kuhlii*). These results increase our knowledge about the transmission mechanisms of adenoviruses.

## Introduction

Some viruses have the potential for cross-species transmission, with spillover episodes from humans to wildlife and *vice versa*, a phenomenon referred to as zoonosis [1]. Current research on the dynamics behind host/virus interactions and inter- and intra-specific transmissions is of scientific interest and important implications for emerging zoonoses and consequently for public health.

The heterogeneity (understood as the inter- and intra-specific variation) in the ability of hosts to transmit pathogens is among the most fundamental concepts in disease dynamics and crucial in the design of disease control strategies [2]. The potentiality of cross-species or cross-populations transmission depends on the characteristics of host and virus traits and is affected directly or indirectly by a variety of ecological, social, or socioeconomic changes [3] that can lead to new conditions boosting the expansion of the viruses to new host species or populations. Recent studies have revealed that traits such as host phylogenetic relatedness, conservation status, and geographical overlap are critical in the potential for cross-species transmission of viruses [4,5].

The highest proportion of zoonotic viruses in mammals is found in bats, primates and rodents [6]. Specific bat characteristics like their ability to fly, unique among mammals, or their migratory capacity [7], increase their potential role as reservoirs of diseases. Bats are natural reservoirs for many viruses, such as coronavirus, Nipah virus and–apparently- Ebola, related to new emerging diseases that have received attention in the last years due to the potential risk they pose for human pandemic events [8–10] or with important health and economic consequences world-wide, such as rabies outbreaks [11,12]. The capacity to coexist with viruses (in the absence of disease) is probably linked to the bats’ immune-system which seems be different to that of other mammals, [7,13] and related to the development of altered mitochondrial genomes [14]. This pathogen control in bat hosts has favored ancient events of coevolution or parallel evolution [15,16] between bats and viruses.

Additionally, bats are very diverse and form the second largest order of mammals [17]. In fact, bats occupy all kinds of habitats throughout all continents except Antarctica, showing a surprising ecological breadth that implies large variation in trait characteristics either in their morphology: e.g. size variation across species [18]; social behavior: e.g. differences in mating system [19]; or habitat requirements: e.g. roosts preferences from trees to caves [18]. Furthermore, all European bats (used for this study) are insectivorous but use very different foraging strategies. For all these reasons, bats provide a good model system to inspect the correlation of host trait characteristics with the prevalence of viruses.

Adenoviruses (AdVs) are non-enveloped dsDNA viruses with a broad range of vertebrate hosts, such as humans. These viruses have been grouped into five genera [20]: *Mastadenovirus* (mammals), *Atadenovirus* (mammals, birds and reptiles), *Aviadenovirus* (birds), *Siadenovirus* (poultry and amphibians) and *Ichtadenovirus* (fish). Symptoms in humans include respiratory or eye infections and intestinal or digestive illness that rarely lead to mortality [21,22]. Since the first detection of an AdV in a fruit bat from Japan [23], a high diversity of AdVs has been isolated from bats from America, Africa and Asia, all grouped within the genus *Mastadenovirus* [24]. The first AdV in a European bat was isolated from a common pipistrelle (*Pipistrellus pipistrellus*) in Germany [25]. Later, Vidovszky *et al*. [26] obtained partial sequences from as many as 28 presumably new AdVs hosted by 12 different bat species in Germany and Hungary. Iglesias-Caballero *et al*. [16] conducted a country-wide survey in Spain constituting the largest screening for AdVs in bats to date, checking >1,000 individuals belonging to 28 species and focusing not only on the analysis of fecal samples and internal tissues -as in previous studies- but also on the analysis of the oropharyngeal swabs. AdVs are detected in almost half of the Iberian bat species studied, in both feces and in the upper respiratory tract (for the first time in bats), establishing a possible fecal-oral transmission route in two *Pipistrellus pygmaeus*. Moreover, they found a surprisingly high prevalence in this species together with the co-generic *P*. *kuhlii* and the closely related giant noctule, *Nyctalus lasiopterus*, which accounted for the vast majority of viruses detected in the study. These results have presented us with the opportunity to analyze the abiotic factors or biotic traits determining prevalence of AdVs in European bats at two levels: across-species and among individuals within a species.

The analysis of morphological and behavioral traits and presence of AdVs in bats is also useful for the understanding of viral transmission dynamics. In fact, little is known about the transmission mechanisms of AdVs and to the best of our knowledge, no study has focused on transmission in bats. A handful of studies in humans suggest that transmission may need direct contact or at least a droplet spraying (such as those produced by coughing or sneezing) or aerosol [27–29]. The strong host specificity and the parallelism between host and AdVs phylogenies found for bats [16,30] and for primates [31] strongly suggest that cross-species switching of mastadenovirus are not frequent events although they have been detected in AdVs evolution [30].

To our knowledge, no study has focused so far on the analyses of the determinant factors influencing prevalence of viruses on bats at both species and individual levels. Across species, Webber *et al*. [32] found that viral richness was positively correlated with group size as predicted by the contact-rate hypothesis. In turn, at the intra-specific level Dietrich *et al*. [33] found an important seasonal shift in prevalence with a significant increase in AdVs shedding during reproduction while studying AdVs prevalence in two bats (*Miniopterus natalensis* and *Rousettus aegyptiacus*). The aim of this study is to advance our understanding of heterogeneity in the prevalence of AdVs in bats, testing the association between traits and presence of AdVs at both the among-species and within-species levels. Among species, and according to the contact-rate hypothesis, higher prevalence of AdVs is expected in species roosting in large groups, sharing refuge with other species and/or mating in swarming aggregations. In addition, we hypothesize a strong phylogenetic signal given the strong species-specificity found in the presence of AdVs. Within species, we predict a higher prevalence in females because of their concentration in large numbers in nursery colonies [34], with high contact rate and a high concomitant risk of infection.

In summary, our aims were to: 1) Test for significant phylogenetic component to the presence of AdVs in European bats; 2) test the importance of ecological characteristics at a species-level for the presence of AdVs taking into account phylogenetic relationships; 3) investigate whether some individual characteristics are determinant to explain the differences in the prevalence of AdVs within species.

## Materials and methods

### Ethics statement

Non-lethal sampling was based on permits 201710730002961/IRM/MDCG/mes issued by Dirección General de Gestión del Medio Natural y Espacios Protegidos (Consejería de Medio Ambiente, Junta de Andalucía, Spain), 10/085545.9/17.9/17 issued by ‘Consejería de Medio Ambiente, Administración Local y Ordenación del Territorio, Comunidad de Madrid’, and PNSNG_SG_2018_0093 issued by ‘Servicio Territorial de Medio Ambiente de Segovia, Junta de Castilla y León’. For this study, permits for the collection of punch biopsies of wing membranes from bats were issued by Comité de Ética del Consejo Superior de Investigaciones Científicas and by Consejería de Agricultura y Pesca y Desarrollo Rural, Junta de Andalucía (permit reference: SSA/SSI/MD/ps). The techniques used meet the guidelines published by the American Society of Mammalogists on the use of wild mammals in research [35]

### Data collection

Most of the information used in this study was obtained during a surveillance program for bat rhabdovirus and lyssavirus carried out between 2004 and 2008, 2016 and 2018 across 21 Iberian localities. Bats were mist-netted near roosts or over water and released at the same collection point after sampling. Each animal was identified, sexed, measured and weighed. Sampling consisted of a 2 mm Ø membrane wing-punch, saliva with oropharyngeal swabs and stool samples (when this was possible). Swabs were stored in 1.5 mL tubes filled with lysis buffer. All samples were aliquoted and stored at -80 °C prior to analysis. Samples were screened for AdVs following published protocols [16,36,37]. Our own database of bats captured in Iberia was combined with the published data of AdVs presence in other bats from Germany and Hungary [25,26]. The identification of bats belonging to species complexes was confirmed through PCR amplification of a diagnostic mtDNA fragment following Ibañez *et al*., [38] and Kaňuch *et al*. [39].

### Phylogeny and phylogenetic signal

A fully resolved phylogeny containing all the study species was not available from the literature and is difficult to reconstruct for this group, [40] so a hybrid approach was taken whereby, published trees were grafted to obtain a full-solved topology whereas branch lengths were estimated using molecular sequence data. The published phylogenetic studies for the four families existing in Europe, used to manually construct a topology of all species in this study were Guillén-Servent *et al*., [41] for relationship in the family Rhinolophidae; Ruedi *et al*., [42] and Stadelmann *et al*. [43] for the subfamily Myotinae within Vespertilionidae; and Hoofer and Bussche [44] for the rest of this last family and for the family Miniopteridae. Complete sequences of the mitochondrial genes *cytochrome b* (*CYTB*) and *NADH dehydrogenase 1* (*ND1*) were obtained from GenBank for all available European species and were used to estimate branch length in the constructed topology. This dataset needed to generate sequences *de novo* for the species *Miniopterus schreibersii* for which these markers were not available. The new sequences are deposited in GenBank under the accession numbers MK737740 and MK737741. For each locus sequences were aligned with the software ‘ALTER’ [45] and MEGA [46], and partitionfinder v2.1.1 [47] was run on the concatenated alignment (1805 base pairs in length) to obtain the optimal partitioning scheme and substitution models. Partitionfinder was set to use unlinked branch lengths, search only BEAST models with the ‘greedy’ algorithm and AICc as the model selection criterion. A maximum of six possible partitions were allowed; the three codon positions of the two loci. BEAST v2.4.7 [48] was then fed the alignment and the fixed topology, and using a relaxed clock log normal model with exponential priors on the mean (mean = 10) and standard deviation (mean = 0.33), was allowed to estimate the branch lengths over 20 million MCMC iterations, storing every 1000th. Chain diagnostics was performed using Tracer v1.7 [49] to ensure sufficient mixing and parameter convergence and a maximum clade credibility tree was generated using TreeAnnotator v.2.4.7 (part of BEAST package) using a 10% burn-in and median node heights.

Fritz’s *D*, Pagel’s *λ* and Blomberg’s *K* parameters were used to estimate the strength of the phylogenetic signal affecting the pattern of AdVs presence across European bats. Fritz’s *D* was used for binary data (presence/absence) so that values close to one indicate that the distribution of the binary trait is random with respect to the given phylogeny and values close to zero indicate the trait is distributed as expected under a Brownian motion model of evolution [50]. When the variable estimated was the proportion of infected bats in each species, Pagel’s *λ* and Blomberg’s *K* parameters were used. Values of *λ* range from zero to one where *λ* = 0 indicates that related species do not share similar values for the trait (percentage of infection) and *λ* = 1 indicates a pattern fully explained by the phylogenetic relationships under Brownian motion, with related species showing similar values for the given trait. Finally, *K* is scaled so that zero indicates no phylogenetic signal, *K* = 1 is the expected value for trait evolution under Brownian motion and values higher than one suggest stronger phylogenetic signal than predicted by Brownian motion [51,52].

### Variables selection and modeling

Ecological and behavioural traits were selected considering their ‘*a priori’* importance for the virus transmission and their availability for the European bat species from the bat literature and general revisions [53], identification guides [34] (S1 Table). For the comparative analysis across all European bats, the following variables were recorded for each species: (1) ‘Group size’, defined as the upper bound of individuals found usually in summer roosts; (2) ‘Forearm’: defined as the average range of forearm length (in mm) reported for each species; (3) ‘Sociability’ defined as the chance of sharing summer roost with other species and considering just two categories: species that have never been found sharing their roost with other species and species that sometimes or always share roost with other species; (4) ‘Mating strategy’: considered as whether mating takes place in seasonal swarming or not; (5) ‘Migration’: defined as whether the bat species is known to perform long-distance seasonal movements (longer than 100 km), regional (between 10 and 100 km) or no movements; (6) ‘Roost Type’: considered in three categories: roosts in caves, trees or crevices. At the individual level, the analyses focused on the three species that showed the highest AdVs presence combined with a large enough sample size (*Nyctalus lasiopterus*: N = 234; *Pipistrellus pygmaeus*: N = 298; *P*. *kuhlii*: N = 230). The recorded variables were: (1) ‘Locality’: site of capture; (2) ‘Sex’: male or female; (3) ‘Forearm’: measured in mm.

For among-species comparisons, AdVs prevalence was analyzed as the percentage of positive samples for each species. The percentage was logit-transformed to conform to the normality assumption of the data. Species with fewer than 10 samples were eliminated from the matrix and from the phylogeny. The analyses were performed with R statistical computing packages [54] and scripts are available as Supplementary Material. Fritz’s D, Pagel’s λ and Blomberg’s K parameters were estimated with the packages ‘*caper*’ [55] and ‘*phytools*’ [56]. Phylogenetic linear regressions were carried out with the package ‘*phylolm*’ [57] that respects the shared evolutionary histories of species [58]. Models of trait evolution were compared assuming correlation structures under either Brownian motion, Ornstein-Uhlenbeck or Pagel models [59] and the best model was selected based on the Akaike Information Criterion (AIC) [60]. As a threshold value for model selection, a model showing ΔAIC > 3 was taken as having greater support [61]. The best correlation structure selected was included in the phylogenetic linear regression, in which the variables aforementioned were selected by considering all possible subsets, again based on the Akaike Information Criterion and with the same threshold value, using the package ‘*MuMIn*’ [62]. Complementary, the analyses were also run considering presence/absence of AdVs as a dependent variable and including in this case all sampled species. A binary phylogenetic generalized linear model was run using the ‘*ape*’ package, with presence of AdVs as the dependent variable [63].

**Fig 1:**
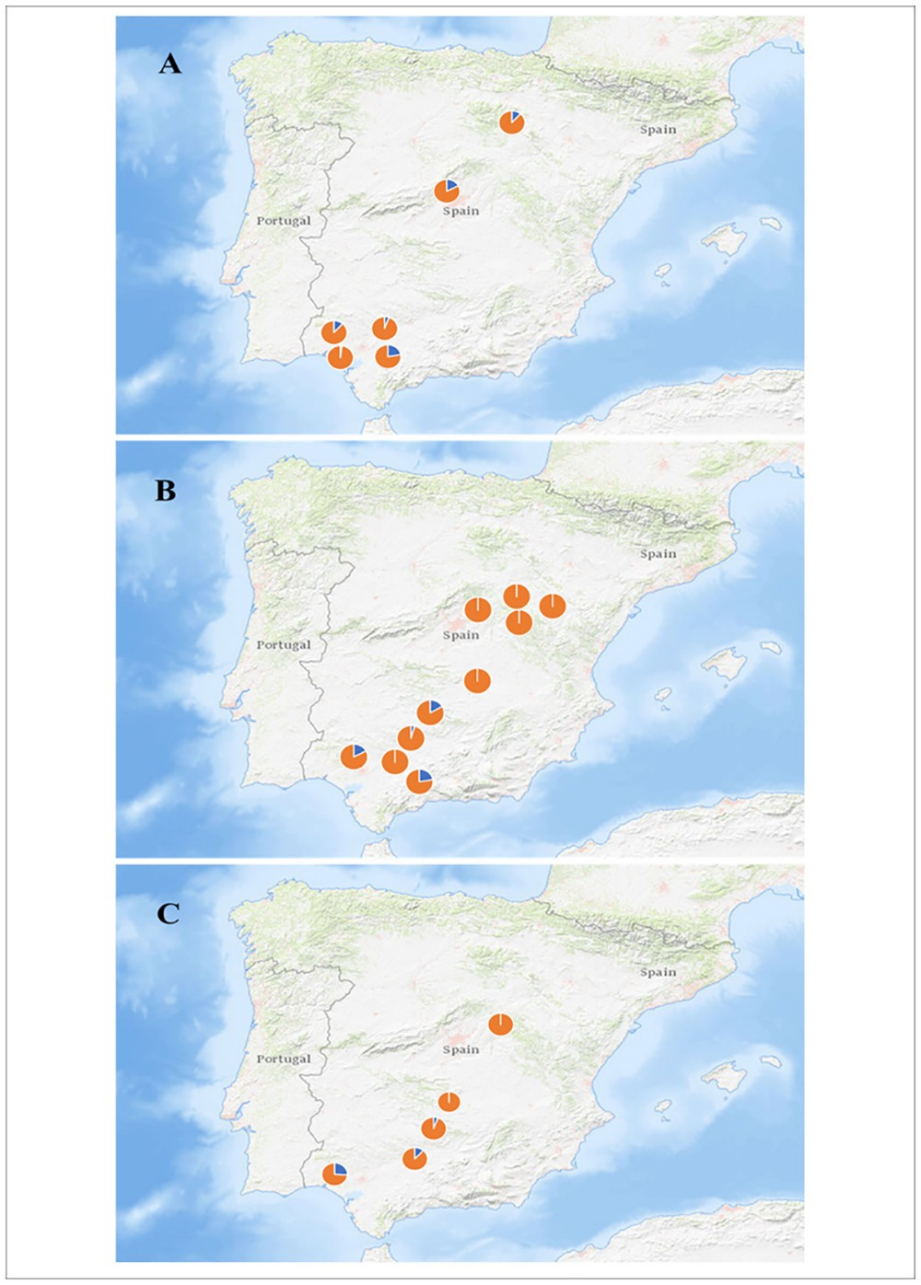
Sampling localities with percentage of AdVs positive samples. Portion of AdVs positive sample is indicated in blue, AdVs negative samples in orange for (**A**) *Nyctalus lasiopterus*, (**B**) *Pipistrellus pygmaeus* (**C**) *P*. *kuhlii*. Only localities with at least 10 recorded individuals are shown. (Modified from USGS National Map Viewer—Public Domain).

Within species, again, we considered only the three species showing the highest AdVs presence combined with a large enough sample size (*Nyctalus lasiopterus*: N = 234; *Pipistrellus pygmaeus*: N = 298; *P*. *kuhlii*: N = 230; distribution of sampling localities shown in Fig 1). We used general linear mixed models implemented in the ‘*lme4*’ package [64] to test for associations between traits and presence of AdVs for each species considered, and considering AdVs presence as a binary dependent variable while ‘Forearm’ and ‘Sex’ were set as independent variables. In order to account for uncontrolled spatial variation, ‘Locality’ was included in the models as a random variable. As a confirmatory analysis for the models, the variance explained by only the fixed variables was compared to the variance obtained including the random variables using the package ‘*MuMIn*’ [62]. At both among-species and within-species levels, inter-correlation was were checked using a Pearson’s correlation test for continuous variables and an ANOVA for categorical variables. The spatial distribution of the AdVs positive bats was inspected by performing a Mantel test between the matrix of geographic distances and the matrix of differences in proportion of infection between sites with the package ‘*ade4*’ [65,66].

## Results

**Fig 2:**
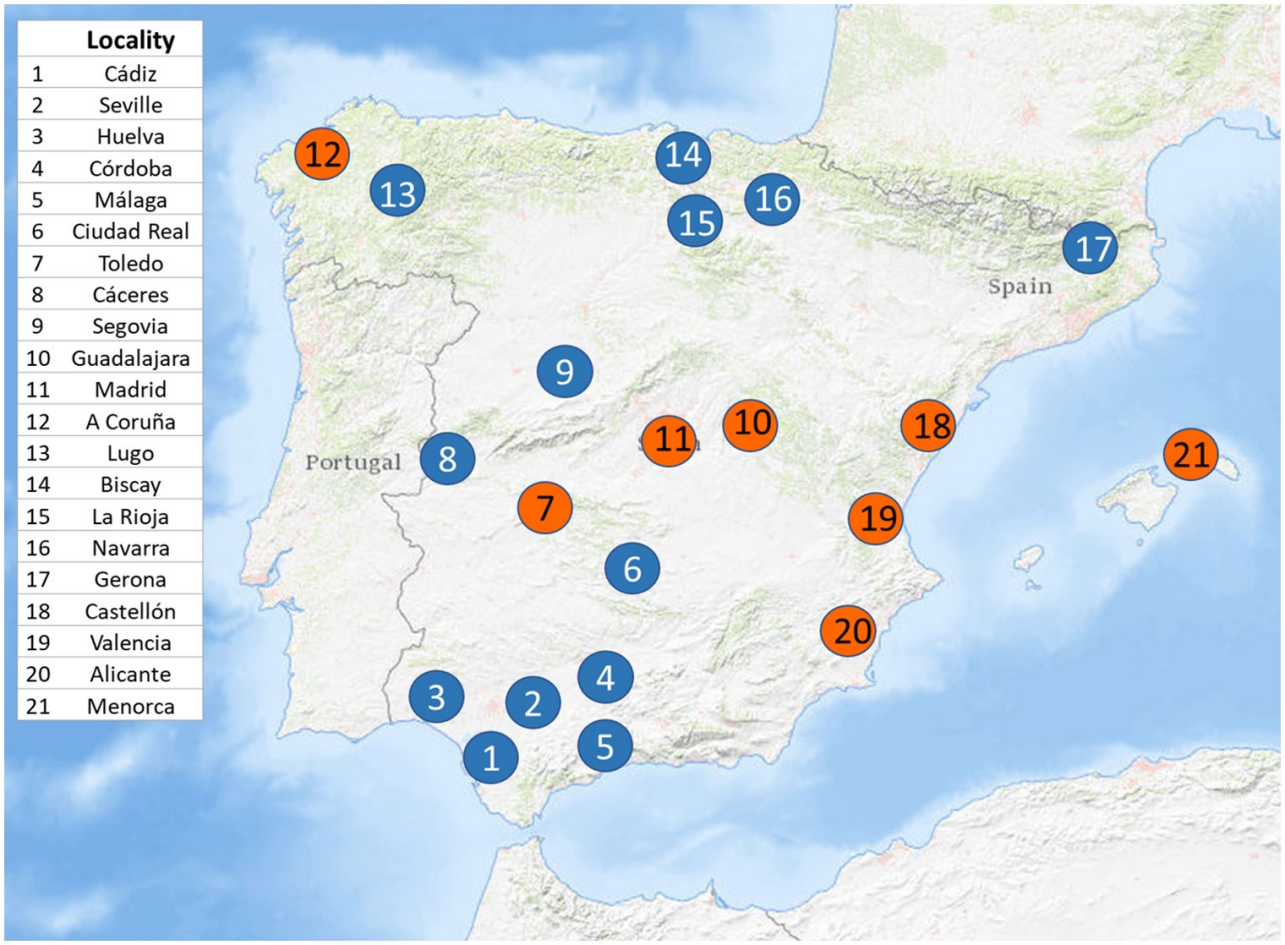
Study sites in Spain where bats were screened for AdVs. Blue dots indicate localities with at least one AdVs positive samples, orange dots indicate localities with negative samples for AdVs. (Modified from USGS National Map Viewer—Public Domain).

The final working database consisted of 1,806 bats sampled and checked for AdVs, belonging to 10 genera (*Barbastella*, *Eptesicus*, *Hypsugo*, *Miniopterus*, *Myotis*, *Nyctalus*, *Pipistrellus*, *Plecotus*, *Rhinolophus*, *Vespertilio*) and representing 33 of the 45 European bat species. The database of bats sampled in the Iberian region included a total of 1,466 bats belonging to 27 Iberian species surveyed for the study (Fig 2). The inclusion in the analyses of the published results from Vidovszky *et al*. [26] and Sonntag *et al*. [25] for Germany and Hungary, allowed the addition of six European species (*Eptesicus nilssonii*, *Myotis brandtii*, *M*. *dasycneme*, *M*. *nattereri*, *Pipistrellus nathusii* and *Vespertilio murinus*), not found in Iberia and therefore not included in the Iberian database.

The number of individuals sampled within species varied considerably and 5 species (*Myotis daubentonii*, *Miniopterus schreibersii*, *Nyctalus lasiopterus*, *Pipistrellus kuhlii*, *P*. *pygmaeus*), accounted for 50% of the total number of individuals sampled for this study. On the other hand, the species *Eptesicus nilssonii*, *Myotis alcathoe*, *M*. *brandtii*, *M*. *crypticus*, *Rhinolophus mehelyii* were represented by fewer than 10 individuals and were consequently discarded from the proportion-based analyses of prevalence. Values of proportion of positive samples varied considerably across species, averaging 7.02% (calculated as the average of the percentages of all the species) (Table 1). The species targeted for the individual-level analyses based on the highest prevalence of AdVs and large enough sample size were: *Nyctalus lasiopterus* (10.26% prevalence), *Pipistrellus pygmaeus* (10.07% prevalence) *and Pipistrellus kuhlii* (9.57% prevalence).

**Table 1:** Number of individuals screened as positive or negative for adenoviruses and percentage of positives for each species taken into account in this study.

Species	N° Positives	N° Negatives	Percentage (%)	Lower limit (%)	Upper limit (%)
*Barbastella barbastellus*	0	46	0.00		
*Eptesicus nilssonii*	1	5	16.67	0.85	58.18
*Eptesicus isabellinus*	4	18	18.18	6.46	36.91
*Eptesicus serotinus*	2	20	9.09	1.64	25.95
*Hypsugo savii*	3	50	5.66	1.56	13.98
*Miniopterus schreibersii*	0	163	0.00		
*Myotis alcathoe*	0	4	0.00		
*Myotis bechsteinii*	1	38	2.56	0.13	11.60
*Myotis blythii*	1	35	2.78	0.14	12.51
*Myotis brandtii*	0	7	0.00		
*Myotis capaccinii*	0	15	0.00		
*Myotis crypticus*	0	3	0.00		
*Myotis dasycneme*	2	3	40.00	7.64	81.07
*Myotis daubentonii*	0	102	0.00		
*Myotis emarginatus*	4	59	6.35	2.20	13.94
*Myotis escalerai*	0	23	0.00		
*Myotis myotis*	5	88	5.38	2.14	10.97
*Myotis mystacinus*	1	29	3.33	0.17	14.86
*Myotis nattereri*	0	13	0.00		
*Nyctalus lasiopterus*	24	210	10.26	7.17	14.12
*Nyctalus leisleri*	5	40	11.11	4.48	21.95
*Nyctalus noctula*	24	60	28.57	20.55	37.77
*Pipistrellus kuhlii*	22	208	9.57	6.56	13.38
*Pipistrellus nathusii*	2	13	13.33	2.42	36.34
*Pipistrellus pipistrellus*	10	85	10.53	5.82	17.20
*Pipistrellus pygmaeus*	30	268	10.07	7.34	13.41
*Plecotus auritus*	1	26	3.70	0.19	16.40
*Plecotus austriacus*	0	15	0.00		
*Rhinolophus euryale*	7	40	14.89	7.20	26.16
*Rhinolophus ferrumequinum*	9	95	8.65	4.59	14.62
*Rhinolophus hipposideros*	1	10	9.09	0.47	36.44
*Rhinolophus mehelyi*	0	1	0.00		
*Vespertilio murinus*	1	14	6.67	0.34	27.94

Data from Vidovszky *et al*. [26] and Sonntag *et al*. [25] included. Lower and upper limits of 95% confidence intervals of the percentage have been included [67].

### Phylogenetic signal

The fully resolved phylogeny for all the studied species with proportional branch lengths was used to estimate the different phylogenetic component in AdVs presence (S1 Fig). Fritz’s *D* parameter was 0.482, and we detected a marginally significant departure from Brownian motion structure (*p* = 0.058), whereas the probability of *D* resulting from Brownian motion structure was *p* = 0.165. The value of Pagel’s *λ* when analyzing the distribution of proportion of AdVs across the phylogeny was *λ* = 0.94 and Blomberg’ *K* was *K* = 0.70. Pagel’s *λ and* Blomberg’ *K* were both significantly different from zero (*λ*: *p* = 0.0003; *K*: *p* = 0.001). Thus, the distribution of the AdVs infection across the studied European bats showed a strong phylogenetic component following Brownian motion.

### Trait association with AdVs prevalence

**Fig 3:**
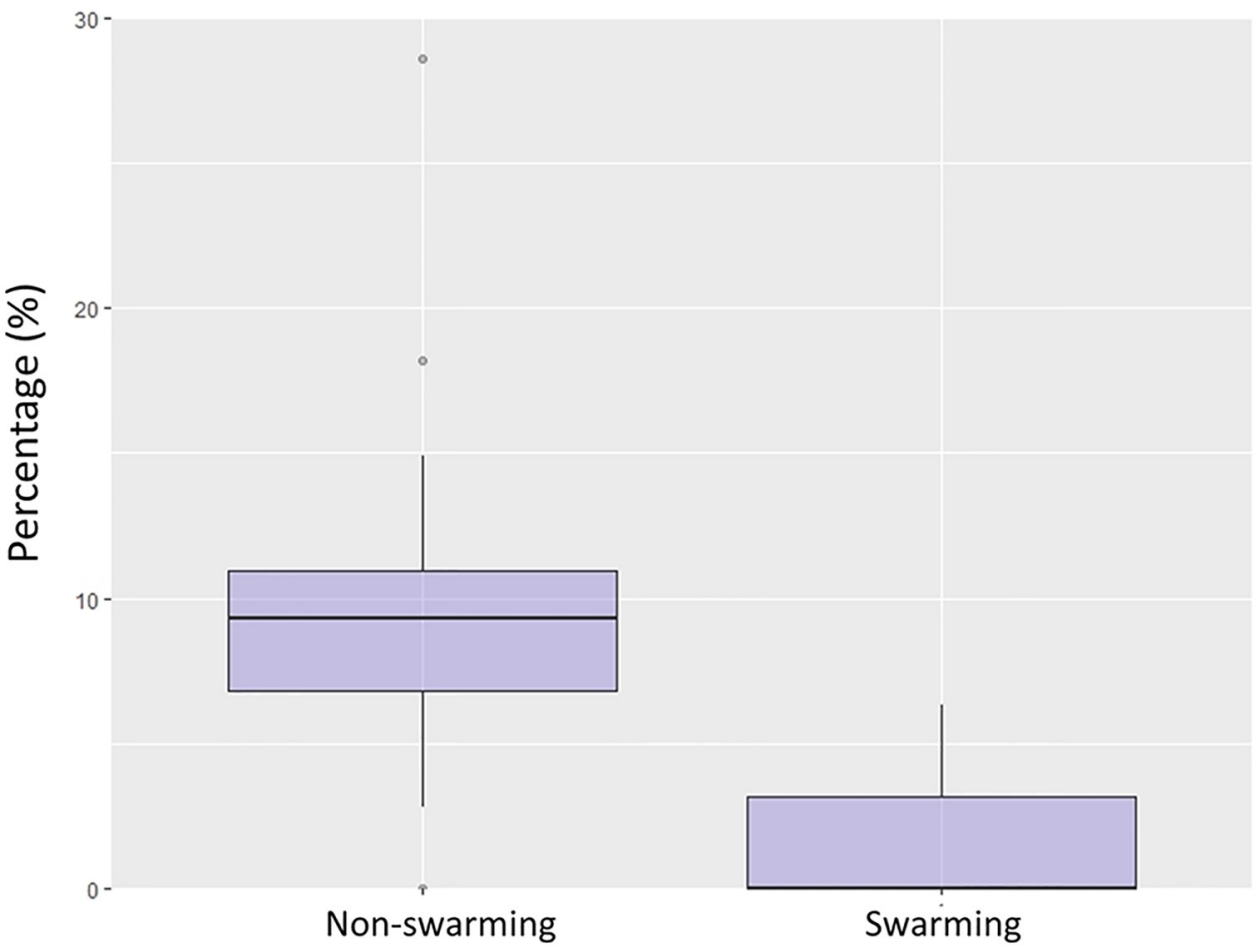
Boxplot of the percentage of positive individuals for species engaging in swarming and species not engaging in swarming. *N* positive species = 14; *N* negative species = 19. Black horizontal lines indicate the median value. Lower and upper hinges correspond to the first and third quartiles. The upper whisker extends from the hinge to the largest value no further than 1.5 * IQR from the hinge (where IQR is the inter-quartile range). The lower whisker extends from the hinge to the smallest value at most 1.5 * IQR of the hinge. Dots beyond the end of the whiskers indicate outlying points.

The phylogenetic linear regression analysis showed that regardless of the phylogenetic model used (although Ornstein-Uhlenbeck had the lowest AIC score, all three models tested were within ΔAIC ≤ 3), the variable ‘Mating strategy’ was selected in the three best models through the Akaike Information Criterion by considering all possible subsets (model selection presented in S2 Table) and showed a significant (and negative) effect, as shown in Fig 3 (model = Ornstein-Uhlenbeck, *t* = -3.5849, *p* = 0.002; all model results presented in S3 Table). Moreover, the same result was recovered when using AdVs presence as a binomial dependent variable (S4 Table), indicating a significantly higher AdVs presence in bat species not engaging in swarming for mating.

**Fig 4:**
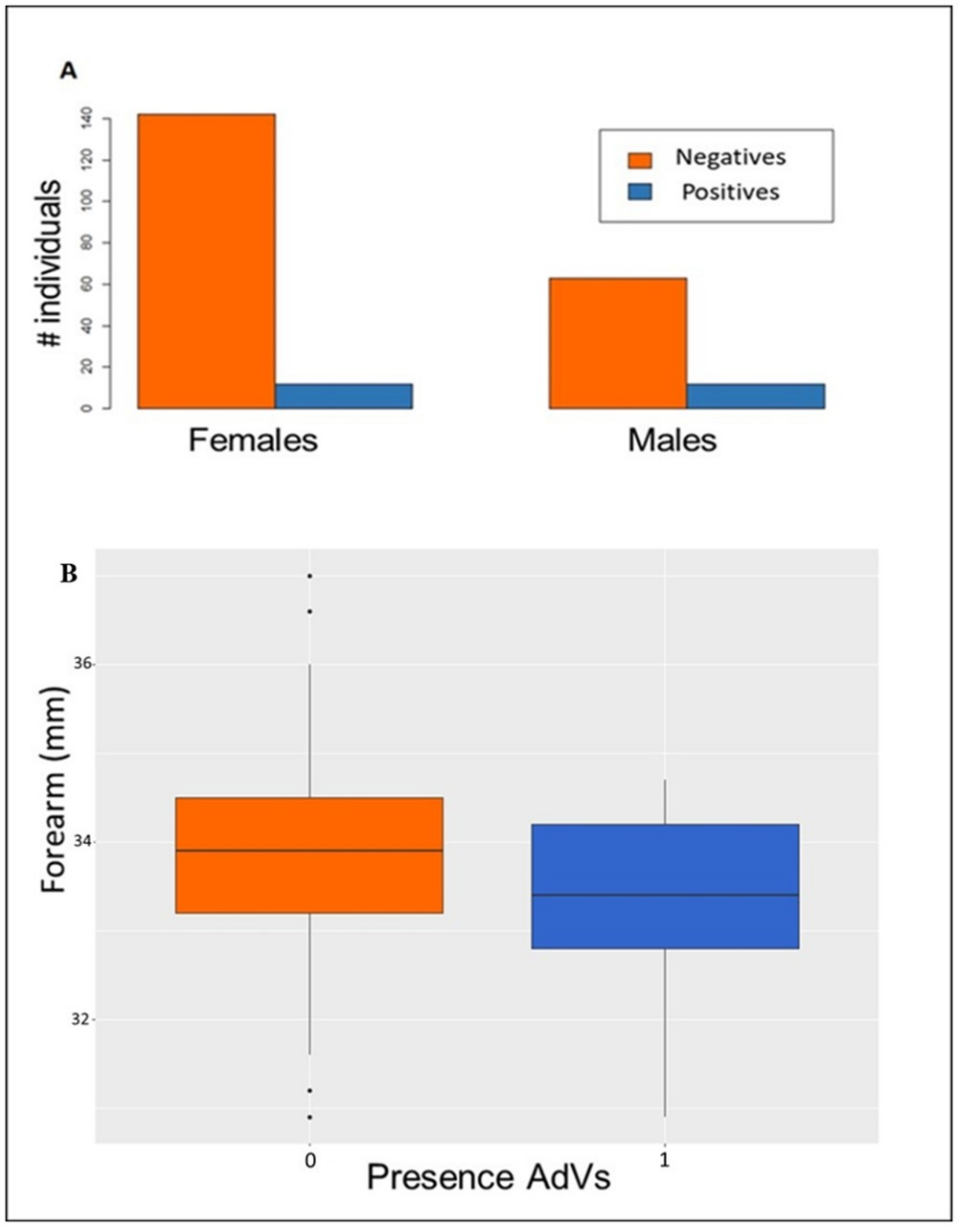
Results of within-species analysis. (**A**) Barplot by sex of number of *Nyctalus lasiopterus* individuals positive (blue) and negative (orange) to adenovirus. (**B**) Boxplot for individuals screening negative for AdVs (0) and positive for AdVs (1) in relation to forearm length for *Pipistrellus kuhlii*. Black lines indicate the median value. Lower and upper hinges correspond to the first and third quartiles. The upper whisker extends from the hinge to the largest value no further than 1.5 * IQR from the hinge (where IQR is the inter-quartile range). The lower whisker extends from the hinge to the smallest value at most 1.5 * IQR of the hinge. Dots beyond the end of the whiskers indicate outlying points.

At individual level and for the three species considered the GLMM analysis revealed a significantly higher AdVs presence for males (*z* = 2.067, *p* = 0.039) in *N*. *lasiopterus* (Fig 4A), and a trend towards higher AdVs prevalence for individuals with smaller forearm (Fig 4B) for *P*. *kuhlii* (*z* = -1.656, *p* = 0.098). The variance of the model explained by only the random variable was 4% for *N*. *lasiopterus*, 62% for *P*. *pygmaeus* and 16% for *P*. *kuhlii*. Results for all the variables are available in supplementary material S5 Table.

### Mantel test

The Mantel tests showed no significant correlation for all pairwise comparisons between geographic distances and differences in the proportion of AdVs presence across localities for *N*. *lasiopterus* (*r* = -0.2873; *p* = 0.909) (S2 Fig), but this correlation was significant for *P*. *pygmaeus* (*r* = 0.3185; *p* = 0.036) and for *P*. *kuhlii* (*r* = 0.6137; *p* = 0.05) indicating that with greater distance, there will be a greater difference in the proportion of individuals affected by AdVs (S2 Fig).

## Discussion

For the first time to our knowledge, adenovirus prevalence and its correlation with host traits is studied across a wide range of bats both at the species and individual level. The understanding of which and how host traits affect the presence of viruses in bats is a key step to the understanding of the transmission mechanisms and evolutionary strategies of viruses. Such mechanisms are key in the process of host switching that cause the appearance of emerging diseases and therefore its understanding is important to improve disease managements [68]. Our results show that adenoviruses seem to be quite common in European bats since they were found across all the studied tribes and in most genera (except for *Barbastella* and *Miniopterus*) but their prevalence varied considerably among species [16]. The three species best sampled and showing the highest frequency of adenovirus infection (*Nyctalus lasiopterus*, *Pipistrellus pygmaeus* and *P*. *kuhlii*) experienced an average of 9.97% prevalence. In contrast, other species showed very low adenovirus presence and was completely absent in others despite their large sample sizes (e.g.: *Miniopterus schreibersii)* but in other species, the absence of positive results could be due to the low number of sampled individuals (generally the rarest bats in Europe).

Fritz’s *D*, Pagel’s *λ* and Blomberg’s *K* parameters all pointed to a phylogenetic signal in the presence of AdVs across European bats and consequently, the need for accounting for phylogenetic relationships in all subsequent models. This phylogenetic component of the distribution pattern of AdVs in bats was also recently suggested by Iglesias-Caballero *et al*. [16] as the sequences of new mastadenoviruses were clustering generally in agreement with the host bat families or even with the bat species.

Contrary to a previous study on the factors influencing viruses on bats [32], we have not found significant effect of the bats’ group size on the presence of adenoviruses for European species. In their study, Webber *et al*. [32] had a wider perspective and focused on overall viral richness in bats, whereas this study is centered exclusively on AdVs and so our differing results may indicate that adenoviruses use different transmission pathways than other viruses. Transmission of respiratory AdVs in humans requires close contact although it can possibly occur through droplet spray or aerosols, but data are still limited [27–29]. In general, little is known about transmission mechanisms of adenoviruses and to our knowledge, no study has focused on this particular aspect across bats, although the host species specificity found for most of the adenoviruses in bats [16] points to cross-species contacts as rare events.

Counterintuitively, species engaging in swarming behavior were found to have significantly lower prevalence of adenoviruses than other bats despite having theoretically greater chances of contact. Swarming related to mating is shown mainly by forest species and is described as the gathering of bats, commonly in caves and underground sites, during a few hours after dusk and for a few days [69]. Two hypotheses could explain this finding. Firstly, bat species engaging in swarming seem to be in close contact for only a short period of time, which would imply a lower chance of transmission compared to bats with polygynous mating systems, like harems, which stay in contact for longer periods of time, sometimes all year around [70]. A second plausible hypothesis is based on the fact that mammals with high infection risk, especially those with promiscuous behavior, develop a stronger immune system compared to species with low infection risk [71,72]. Thus, it seems reasonable that bats with swarming behavior may show stronger immune response protecting them from infections, despite being very costly and even represent a trade-off with other life-history traits [72].

When analyzing the effect on the AdVs presence of within-species variance in traits, we found that the site of capture explains an important part of the variance of all models suggesting an underlying general pattern across species and supporting the contact-rate hypothesis. This trend is particularly clear for *Pipistrellus pygmaeus* and for *P*. *kuhlii*, whose sampling localities are more widespread along the Iberian Peninsula than those for *Nyctalus lasiopterus* (Fig 1). Following the contact-rate hypothesis, bats from a specific locality interact with other individuals of the same area, favoring the virus transmission locally [7]. A similar pattern was already found for the viruses responsible for avian influence, Marburg or Ebola [73–75]. In this direction, the Mantel’s test supported a significant geographic component in the incidence of the adenoviruses for *Pipistrellus pygmaeus* and for *P*. *kuhlii* which are typically sedentary species [34].

A higher prevalence of adenoviruses was found in males for *Nyctalus lasiopterus*, the species in which the different sexes were best represented. This result is particularly interesting since it could affect the general dynamics of the virus given the generally predominant role played by males in dispersal [76]. The possibility of a sex biased infection as a general pattern should be explored further in other bat species and could be related to the trade-off between immune function and reproduction, making males more susceptible to virus infections during reproductive season due to the immune-suppressant effect of testosterone production [77].

Our results show that, individuals with smaller forearm are more likely to be infected by adenoviruses for *Pipistrellus kuhlii*. Subadult bats usually have slightly longer forearms [34] and the lower prevalence in bats with larger measures could be explained by the influence of the antibody loads transferred to them through their mother’s milk in still developing bats, making them less suitable to adenoviruses prevalence [78]. This result could be also associated with the known fact that young bats stay all together in nurseries. As a consequence, the probability of enter in contact with adult infected bats might decrease [34]. Nevertheless, these are just hypotheses and further studies should help understand the relationships between adenoviruses presence and age.

In sum, our findings highlight a common occurrence of adenoviruses in European bats and provide evidence of the importance of mating behavior in the prevalence of adenoviruses, contrary to what has been suggested generally for viruses. Besides, the three European bats species that had higher adenovirus prevalence do not show any common pattern, pointing to adenoviruses transmission as a complex process. Our study highlights the importance of combining behavioral with ecological traits in explaining viral richness and transmission.

## Supporting information

S1 Table Traits used in the model for each species. Information taken from: 1: Dietz *et al*.[34], 2: Action Plan for the Conservation of All Bat Species in the European Union [53]. Values indicated with asterisk have been obtained by personal communication of Jens Rydell and Jesús Nogueras Montiel. For the variable ‘Refuge’: 1, 2, 3 indicate respectively whether the species roosts in caves, trees or crevices. ‘Swarming’: 0 indicates species not engaging in swarming behavior, 1 indicates species engaging in swarming behavior. ‘Sociability’: 0 indicates species that never share the roost with other species, 1 indicates species sometimes or always share the roost with other species. ‘Migration’: 0 indicates species not performing seasonal movement or performing no movement, 1 indicates species performing long-distance movements.
(DOCX)

S2 Table Model selection of the percentage models under Brownian motion, Pagel and Ornstein-Uhlenbeck structures. Degrees of freedom (df), log likelihood (LogLik), AICc, ΔAIC (Delta) and AIC weight (w) included. Only models with ΔAIC < 4 are shown. Variables selected are indicated as: MS for ‘Mating strategy’. S for ‘Sociability’. F for ‘Forearm’. GS for ‘Group size’. R for ‘Roost type’. M for ‘Migration’.
(DOCX)

S3 Table Result of the percentage models. Variables selected in the three best models under Akaike Information Criterion are shown under Brownian motion, Pagel and Ornstein-Uhlenbeck structures.
(DOCX)

S4 Table Results of absence/presence model. Estimate value, standard error, Z-score and p-value included.
(DOCX)

S5 Table Result of GLMM model (individual level) for each species taken into account. A: *Nyctalus lasiopterus*. B: *Pipistrellus pygmaeus*. C: *Pipistrellus kuhlii*.
(DOCX)

S1 Fig Evolutionary relationships of bat species considered in this study. Based on the phylogenetic hypotheses of: Guillén-Servent *et al* [41].; Hoofer and Bussche [44]; Ruedi *et al*. [42] and Stadelmann *et al*. [43]. Branch lengths reflect mitochondrial sequence divergences. Tip labels indicate species screening positive (black) or negative (white) for AdVs.
(TIF)

S2 Fig Scatterplot showing the results of the Mantel test. Mantel test between the matrix of differences in percentages of AdVs presence and the matrix of geographic distances for (A) *Nyctalus lasiopterus* (B) *Pipistrellus pygmaeus* and (C) *P*. *kuhlii*. Geographic distances are calculated as Euclidian distances. Only localities with at least 10 individuals sampled have been taken into account.
(TIF)

S1 File Raw data with information about each individual. “Locality”, “Date of capture” and “Colony” indicates respectively locality, date and colony of capture and the locality or organism sending the samples to be analyzed. “ID” indicates Identification number, “Sex” indicates whether male (‘m’) or female (‘h’). Column “AdV” indicates whether the individual resulted positive (‘POSITIVO’) or negative (‘NEGATIVO’) to Adenoviruses.
(CSV)

S2 File Newick format of the phylogeny of bat species considered. (TRE)

S3 File R script containing R codes used to perform the analyses. (TXT)
