# Supplementary material for: Mating strategy is determinant of adenovirus prevalence in European bats
Source: PLoS One. 2020 Jan 7;15(1):e0226203. doi: 10.1371/journal.pone.0226203 (PMC6946596; doi:10.1371/journal.pone.0226203)
Supplement: S1 Table — Information taken from: 1: Dietz et al.[34], 2: Action Plan for the Conservation of All Bat Species in the European Union [53]. Values indicated with asterisk have been obtained by personal communication of Jens Rydell and Jesús Nogueras Montiel. For the variable ‘Refuge’: 1, 2, 3 indicate respectively whether the species roosts in caves, trees or crevices. ‘Swarming’: 0 indicates species not engaging in swarming behavior, 1 indicates species engaging in swarming behavior. ‘Sociability’: 0 indicates species that never share the roost with other species, 1 indicates species sometimes or always share the roost with other species. ‘Migration’: 0 indicates species not performing seasonal movement or performing no movement, 1 indicates species performing long-distance movements. (DOCX) [file pone.0226203.s001.docx]

**S1 Table**

| **Species** | **Group size^1^** | **Forearm (mm)^1^** | | **Sociability^1^** | **Swarming^1^** | **Refuge^1^** | **Migration^2^** |
| --- | --- | --- | --- | --- | --- | --- | --- |
| *Barbastella barbastellus* | 20 | 40 | 0 | | 1 | 2 | 0 |
| *Eptesicus nilssonii* | 50 | 40.65 | 0 | | 0 | 3 | 0 |
| *Eptesicus isabellinus* | 100* | 49 | 0 | | 0 | 3 | 0 |
| *Eptesicus serotinus* | 60 | 53 | 0 | | 0 | 3 | 0 |
| *Hypsugo savii* | 70 | 34.65 | 0 | | 0 | 3 | 0 |
| *Miniopterus schreibersii* | 6000* | 45.2 | 1 | | 0 | 1 | 0 |
| *Myotis alcathoe* | 80 | 32.15 | 0 | | 1 | 2 | 0 |
| *Myotis bechsteinii* | 50 | 43.05 | 0 | | 1 | 2 | 0 |
| *Myotis blythii* | 5000 | 56.75 | 1 | | 0 | 1 | 0 |
| *Myotis brandtii* | 60 | 35.6 | 0 | | 1 | 3* | 0 |
| *Myotis capaccinii* | 500* | 41.2 | 1 | | 1 | 1 | 0 |
| *Myotis dasycneme* | 100 | 46 | 1 | | 1 | 3* | 0 |
| *Myotis daubentonii* | 50 | 37.55 | 1 | | 1 | 3 | 0 |
| *Myotis emarginatus* | 500 | 40.4 | 1 | | 1 | 1 | 0 |
| *Myotis escalerai* | 1000 | 39.8 | 1 | | 1 | 1 | 0 |
| *Myotis myotis* | 1000 | 60.95 | 1 | | 0 | 1 | 0 |
| *Myotis mystacinus* | 60 | 34.25 | 0 | | 1 | 2 | 0 |
| *Myotis nattereri* | 50 | 39.2 | 0 | | 1 | 2 | 0 |
| *Myotis spA* | 50* | 39.2 | 0 | | 1 | 2 | 0 |
| *Nyctalus lasiopterus* | 35 | 65.5 | 0 | | 0 | 2 | 0 |
| *Nyctalus leisleri* | 50 | 43.8 | 0 | | 0 | 2 | 1 |
| *Nyctalus noctula* | 50 | 53.1 | 0 | | 0 | 2 | 1 |
| *Pipistrellus kuhlii* | 100 | 33.85 | 0 | | 0 | 3 | 0 |
| *Pipistrellus nathusii* | 200 | 34.65 | 0 | | 0 | 3 | 1 |
| *Pipistrellus pipistrellus* | 100 | 31.25 | 0 | | 0 | 3 | 0 |
| *Pipistrellus pygmaeus* | 250 | 30 | 0 | | 0 | 3 | 0 |
| *Plecotus auritus* | 50 | 39.15 | 1 | | 1 | 2 | 0 |
| *Plecotus austriacus* | 30 | 40 | 1 | | 1 | 3 | 0 |
| *Rhinolophus euryale* | 1500 | 48 | 1 | | 0 | 1 | 0 |
| *Rhinolophus ferrumequinum* | 1000 | 57.7 | 1 | | 0 | 1 | 0 |
| *Rhinolophus hipposideros* | 50 | 37.85 | 1 | | 0 | 1 | 0 |
| *Rhinolophus mehelyi* | 500 | 51.55 | 1 | | 0 | 1 | 0 |
| *Vespertilio murinus* | 50 | 45.55 | 0 | | 0 | 3 | 0 |
