# Supplementary material for: Mating strategy is determinant of adenovirus prevalence in European bats
Source: PLoS One. 2020 Jan 7;15(1):e0226203. doi: 10.1371/journal.pone.0226203 (PMC6946596; doi:10.1371/journal.pone.0226203)
Supplement: S2 Table — Degrees of freedom (df), log likelihood (LogLik), AICc, ΔAIC (Delta) and AIC weight (w) included. Only models with ΔAIC < 4 are shown. Variables selected are indicated as: MS for ‘Mating strategy’. S for ‘Sociability’. F for ‘Forearm’. GS for ‘Group size’. R for ‘Roost type’. M for ‘Migration’. (DOCX) [file pone.0226203.s002.docx]

| *Brownian motion* |  | |  |  |  |  |
| --- | --- | --- | --- | --- | --- | --- |
| Variables selected | **df** | | **LogLik** | **AICc** | **Delta** | **w** |
| MS | 3 | | 48.260 | -89.5 | 0.00 | 0.356 |
| M + MS | 4 | | 48.858 | -87.9 | 1.58 | 0.162 |
| MS + S | 4 | | 48.647 | -87.5 | 2.00 | 0.131 |
| MS + F | 4 | | 48.551 | -87.3 | 2.19 | 0.119 |
| GS + MS | 4 | | 48.261 | -86.7 | 2.77 | 0.089 |
| S + M + MS | 5 | | 49.290 | -85.7 | 3.75 | 0.055 |
| *Pagel* |  | |  |  |  |  |
| Variables selected | **df** | | **LogLik** | **AICc** | **Delta** | **w** |
| MS | 4 | | 49.586 | -89.4 | 0.00 | 0.415 |
| GS+ MS | 5 | | 50.438 | -88.0 | 1.34 | 0.213 |
| MS + S | 5 | | 50.264 | -87.7 | 1.68 | 0.179 |
| F + MS | 5 | | 49.651 | -86.4 | 2.91 | 0.097 |
| MS + M | 5 | | 49.651 | -86.4 | 2.91 | 0.097 |
| *Ornstein-Uhlenbeck* |  |  | |  |  |  |
| Variables selected | **df** | | **LogLik** | **AICc** | **Delta** | **w** |
| MS | 4 | | 50.067 | -90.3 | 0.00 | 0.408 |
| S + MS | 5 | | 50.900 | -88.9 | 1.37 | 0.205 |
| MS + GS | 5 | | 50.821 | -88.8 | 1.53 | 0.190 |
| MS + F | 5 | | 50.181 | -87.5 | 2.81 | 0.100 |
| M + MS | 5 | | 50.155 | -87.5 | 2.86 | 0.097 |

**S2 Table**
