# Supplementary material for: Mating strategy is determinant of adenovirus prevalence in European bats
Source: PLoS One. 2020 Jan 7;15(1):e0226203. doi: 10.1371/journal.pone.0226203 (PMC6946596; doi:10.1371/journal.pone.0226203)
Supplement: S3 Table — Variables selected in the three best models under Akaike Information Criterion are shown under Brownian motion, Pagel and Ornstein-Uhlenbeck structures. (DOCX) [file pone.0226203.s003.docx]

**S3 Table**

| **Brownian motion** |  |  |  |  |  |
| --- | --- | --- | --- | --- | --- |
| *Model 1* | **AICc = -90.52** |  |  |  |  |
|  | Estimate | Std Error | t.value | p.value |  |
| (Intercept) | 0.074633 | 0.038548 | 1.9361 | 0.06424 | . |
| Mating strategy | -0.055747 | 0.023842 | -2.3382 | 0.02768 | * |
|  | **AICc = -89.72** |  |  |  |  |
|  | Estimate | Std Error | t.value | p.value |  |
| (Intercept) | 0.075392 | 0.038486 | 1.9589 | 0.06184 | . |
| Migration | -0.018859 | 0.018077 | -1.0433 | 0.30722 |  |
| Mating strategy | -0.056614 | 0.023815 | -2.3773 | 0.02576 | * |
| *Model 3* | **AICc = -89.29** |  |  |  |  |
|  | Estimate | Std Error | t.value | p.value |  |
| (Intercept) | 0.086540 | 0.041312 | 2.0948 | 0.04692 | * |
| Sociability | -0.016360 | 0.019560 | -0.8364 | 0.41118 |  |
| Mating strategy | -0.057277 | 0.024056 | -2.3809 | 0.02555 | * |
| **Pagel** |  |  |  |  |  |
| *Model 1* | **AICc = - 91.17** |  |  |  |  |
|  | Estimate | Std Error | t.value | p.value |  |
| (Intercept) | 0.079295 | 0.026563 | 2.9852 | 0.006257 | ** |
| Mating strategy | -0.061025 | 0.020230 | -3.0166 | 0.005802 | ** |
| *Model 2* | **AICc = -90.88** |  |  |  |  |
|  | Estimate | Std Error | t.value | p.value |  |
| (Intercept) | 0.145455 | 0.028354 | 5.1300 | 2.992e-05 | *** |
| Mating strategy | -0.089227 | 0.016268 | -5.4849 | 1.224e-05 | *** |
| Group size | -0.020211 | 0.011534 | -1.7523 | 0.0925 | . |
| *Model 3* | **AICc = -90.53** |  |  |  |  |
|  | Estimate | Std Error | t.value | p.value |  |
| (Intercept) | 0.095080 | 0.026785 | 3.5498 | 0.001629 | ** |
| Sociability | -0.021067 | 0.018249 | -1.1544 | 0.259695 |  |
| Mating strategy | -0.061909 | 0.019609 | -3.1572 | 0.004259 | ** |
| **Ornstein-Uhlenbeck** |  |  |  |  |  |
| *Model 1* | **AICc = -92.13** |  |  |  |  |
|  | Estimate | Std Error | t.value | p.value |  |
| (Intercept) | 0.091506 | 0.013700 | 6.6792 | 5.337e-07 | *** |
| Mating strategy | -0.068957 | 0.019235 | -3.5849 | 0.001426 | ** |
| *Model 2* | **AICc = -91.8** |  |  |  |  |
|  | Estimate | Std Error | t.value | p.value |  |
| (Intercept) | 0.102440 | 0.014154 | 7.2374 | 1.773e-07 | *** |
| Sociability | -0.021888 | 0.017361 | -1.2608 | 0.219512 |  |
| Mating strategy | -0.068525 | 0.018450 | -3.7141 | 0.001082 | ** |
| *Model 3* | **AICc = -91.64** |  |  |  |  |
|  | Estimate | Std Error | t.value | p.value |  |
| (Intercept) | 0.134702 | 0.030289 | 4.4473 | 0.0001694 | *** |
| Mating strategy | -0.083830 | 0.017726 | -4.7292 | 8.268e-05 | *** |
| Group size | -0.016384 | 0.012201 | -1.3428 | 0.1919059 |  |
| Signif. codes: 0 ‘***’ 0.001 ‘**’ 0.01 ‘*’ 0.05 ‘.’ 0.1 ‘ ’ 1 | | | |  |  |
