# Supplementary material for: Mating strategy is determinant of adenovirus prevalence in European bats
Source: PLoS One. 2020 Jan 7;15(1):e0226203. doi: 10.1371/journal.pone.0226203 (PMC6946596; doi:10.1371/journal.pone.0226203)
Supplement: S4 Table — Estimate value, standard error, Z-score and p-value included. (DOCX) [file pone.0226203.s004.docx]

**S4 Table**

|  | Value | Std.Error | Zscore | Pvalue |  |
| --- | --- | --- | --- | --- | --- |
| (Intercept) | -4.30193 | 17.40189 | -0.2472 | 0.80475 |  |
| Group size | -0.45076 | 1.72685 | -0.261 | 0.79407 |  |
| Forearm | 3.60796 | 10.74576 | 0.3358 | 0.73705 |  |
| Mating strategy | -4.64229 | 2.2855 | -2.0312 | 0.04224 | * |
| Migration | 5.59617 | 33.0286 | 0.1694 | 0.86546 |  |
| Sociability | 1.29752 | 2.09474 | 0.6194 | 0.53564 |  |
| Refuge (trees) | 2.36093 | 3.28111 | 0.7196 | 0.4718 |  |
| Refuge (crevices) | 1.06734 | 2.63199 | 0.4055 | 0.68509 |  |
| Signif. codes: 0 ‘***’ 0.001 ‘**’ 0.01 ‘*’ 0.05 ‘.’ 0.1 ‘ ’ 1 | | | | |  |
