# Supplementary material for: Mating strategy is determinant of adenovirus prevalence in European bats
Source: PLoS One. 2020 Jan 7;15(1):e0226203. doi: 10.1371/journal.pone.0226203 (PMC6946596; doi:10.1371/journal.pone.0226203)
Supplement: S5 Table — A: Nyctalus lasiopterus. B: Pipistrellus pygmaeus. C: Pipistrellus kuhlii. (DOCX) [file pone.0226203.s005.docx]

**S5 Table**

| **A** |  |  |  |  |  |
| --- | --- | --- | --- | --- | --- |
|  | Estimate | Std. Error | z value | Pr(>\|z\|) |  |
| (Intercept) | -3.6035 | 0.798 | -4.516 | 6.31E-06 | *** |
| Sex (Male) | 1.0208 | 0.4939 | 2.067 | 0.0387 | * |
| Forearm | 0.4267 | 0.2664 | 1.602 | 0.1092 |  |
| **B** |  |  |  |  |  |
|  | Estimate | Std. Error | z value | Pr(>\|z\|) |  |
| (Intercept) | -3.7204 | 1.0405 | -3.576 | 0.000349 | *** |
| Sex (Male) | 0.4807 | 0.9311 | 0.516 | 0.605683 |  |
| Forearm | 0.1687 | 0.2395 | 0.705 | 0.481094 |  |
| **C** |  |  |  |  |  |
|  | Estimate | Std. Error | z value | Pr(>\|z\|) |  |
| (Intercept) | 11.2906 | 8.5195 | 1.325 | 0.1851 |  |
| Sex (Male) | -15.5999 | 1024.001 | -0.015 | 0.9878 |  |
| Forearm | -0.4159 | 0.2511 | -1.656 | 0.0977 | . |
| Signif. codes: 0 ‘***’ 0.001 ‘**’ 0.01 ‘*’ 0.05 ‘.’ 0.1 ‘ ’ 1 | | | |  |  |
