# Supplementary figures and images for: Mating strategy is determinant of adenovirus prevalence in European bats
Source: PLoS One. 2020 Jan 7;15(1):e0226203. doi: 10.1371/journal.pone.0226203 (PMC6946596; doi:10.1371/journal.pone.0226203)

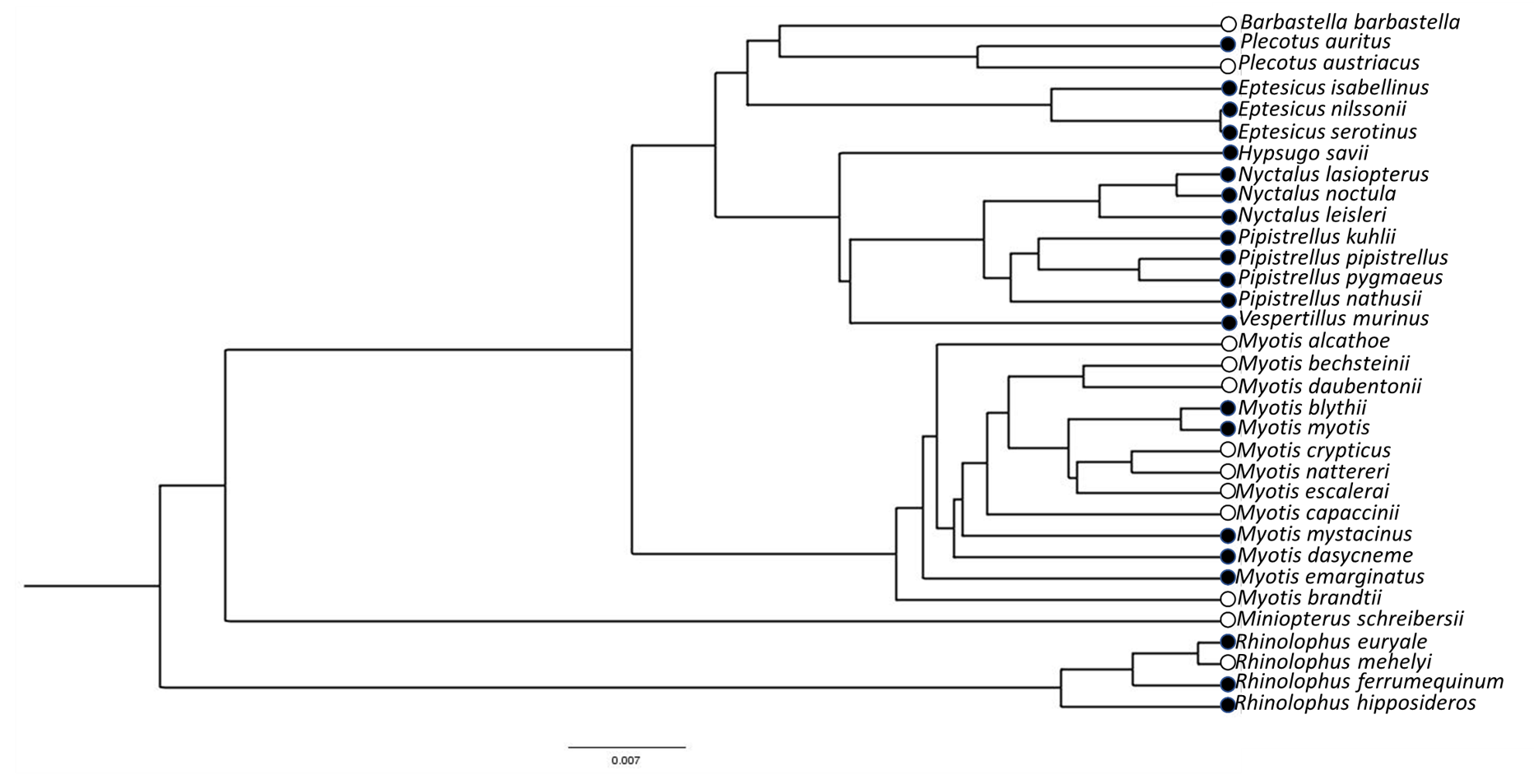

Supplement: S1 Fig — Based on the phylogenetic hypotheses of: Guillén-Servent et al [41].; Hoofer and Bussche [44]; Ruedi et al. [42] and Stadelmann et al. [43]. Branch lengths reflect mitochondrial sequence divergences. Tip labels indicate species screening positive (black) or negative (white) for AdVs. (TIF) [file pone.0226203.s006.tif]

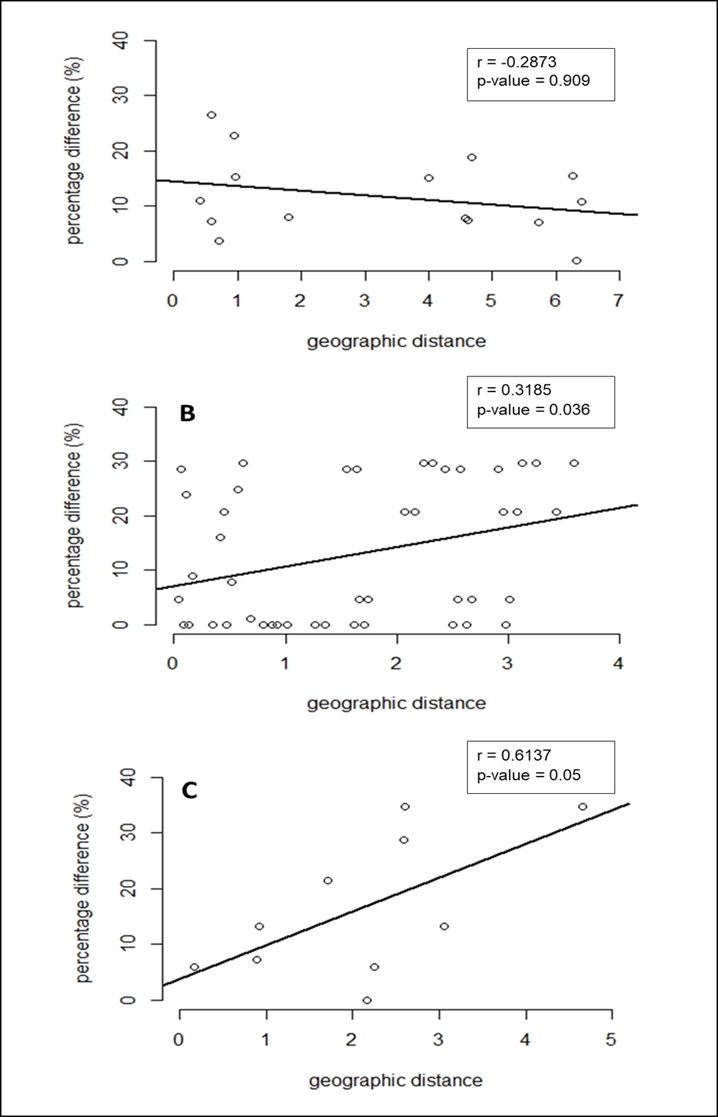

Supplement: S2 Fig — Mantel test between the matrix of differences in percentages of AdVs presence and the matrix of geographic distances for (A) Nyctalus lasiopterus (B) Pipistrellus pygmaeus and (C) P. kuhlii. Geographic distances are calculated as Euclidian distances. Only localities with at least 10 individuals sampled have been taken into account. (TIF) [file pone.0226203.s007.tif]
